# Diagnosis and Management of Acute Pancreatitis

**DOI:** 10.3390/diagnostics15030258

**Published:** 2025-01-23

**Authors:** Nitish Mittal, Veeral M. Oza, Thiruvengadam Muniraj, Truptesh H. Kothari

**Affiliations:** 1 Department of Internal Medicine, The University of Texas Health Sciences Center, Houston, TX 77030, USA; nitishm96@gmail.com (N.M.); veeral_oza@bshsi.org (V.M.O.); 2 Section of Digestive Disease, Edward via College of Osteopathic Medicine and Bon Secours Mercy Health Medical Center, Greenville, SC 29673, USA; 3 Section of Digestive Disease, Yale University School of Medicine, New Haven, CT 06520, USA; thiruvengadam.muniraj@yale.edu; 4 Section of Digestive Disease, University of Rochester Medical Center, Rochester, NY 14642, USA

**Keywords:** acute pancreatitis, diagnosis, management, gallstones, recurrence

## Abstract

Acute pancreatitis is an inflammatory condition of the exocrine pancreas that is a common indication for hospital admission and has had an increasing incidence in the last few decades. The diagnosis of acute pancreatitis requires the satisfaction of two out of three criteria: (1) abdominal pain radiating to the back, (2) serum lipase or amylase levels three or more times the upper limit of the normal level, and (3) findings indicating pancreatitis obtained via a computed tomography (CT) scan or magnetic resonance imaging (MRI). The different etiologies include gallstones, autoimmune disorders, alcohol abuse, smoking, hypertriglyceridemia, obesity, drugs, and post-endoscope retrograde cholangiopancreatography (ERCP). The initial investigation includes serum amylase and lipase analysis, a lipid panel including triglycerides, analysis of immunoglobulins, a full blood count, electrolyte analysis, a hemoglobin A1c test, a complete metabolic panel, and transabdominal ultrasound. The initial therapy includes oxygen supplementation, the provision of intravenous fluids, pain control, and a nutrition regime. Early oral feeding is encouraged if tolerated; if not, liquid supplement provision or enteral tube feeding within 48 h of admission has shown better outcomes. Some complications of acute pancreatitis are necrosis, infection, insulin resistance leading to diabetes mellitus, and pancreatic exocrine insufficiency requiring enzyme supplementation. Patients need to attend regular follow-ups and abstain from alcohol and smoking (if warranted) to prevent the recurrence of acute pancreatitis. The mortality rate of acute pancreatitis has decreased in the past few decades because of better management skills, but the recent rise in acute pancreatitis episodes is concerning. Sustained endeavors through clinical trials are required to establish a broad variety of drugs that can be used for acute pancreatitis episodes.

## 1. Introduction

Acute pancreatitis is an inflammatory condition of the exocrine pancreas that leads to severe abdominal pain, elevated amylase and lipase levels, and/or organ dysfunction. Acute pancreatitis has a global incidence of 30–40 cases per 100,000 people per year [1], while the incidence in children is around 10–15 cases per 100,000 children [2]. These pancreatitis episodes can cause pancreatic necrosis with multiple organ failure, leading to a mortality rate of 1–5% [1]. The average cost of treatment for an acute pancreatitis episode is around USD 10,000 [3]. Some episodes can lead to prolonged hospitalization that can have a significant impact on quality of life due to chronic pain and the corresponding socio-economic burden.

There are more than 80,000 references to acute pancreatitis diagnosis, classification, and management on PubMed, with few studies reporting randomized trials of targeted treatments. Although extensive studies and data collection have been performed, we are still unable to provide an internationally licensed drug therapy that could prove beneficial in the context of pancreatitis. In this article, we review the current state of affairs regarding acute pancreatitis, highlighting the importance of diagnosis in order to determine the etiology and complications of acute pancreatitis, as both can alter inpatient as well as outpatient management.

## 2. Diagnosis

### 2.1. Criteria

**Figure 1:**
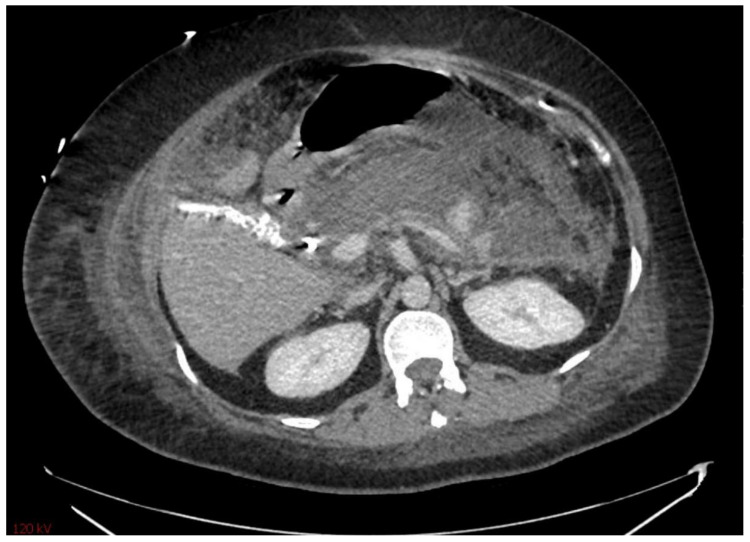
Severe necrotizing pancreatitis.

A diagnosis of acute pancreatitis requires two out of three criteria to be met: (1) abdominal pain radiating to the back, (2) serum lipase or amylase levels three or more times the upper limit of the normal level, and (3) findings indicating pancreatitis obtained via a computed tomography (CT) scan or magnetic resonance imaging (MRI) [4] (Figure 1). We need to be cautious with these criteria, as the first two criteria alone may fail to identify a quarter of patients with acute pancreatitis [5]. One should also be acutely aware of the limitations associated with these imaging modalities. MRI is more expensive than CT scans, whereas CT scans involve the usage of a contrast agent, which has its own risk factors [6]. Furthermore, CT scans also involve ionizing radiation, which can damage the human body. Multiple CT scans over the lifetime of a patient can also have an accumulative impact on the human body. All of these factors should be considered by clinicians prior to deciding on whether to use MRI or a CT scan for the evaluation of AP. The symptoms presented by individuals with pancreatitis include epigastric or diffuse abdominal pain (80–90%), abdominal distension, nausea and vomiting (50–80%), fever, tachycardia, and tachypnea [7]. Because of this wide range of symptoms, physicians need to carefully assess a given patient’s condition and make the correct diagnosis when it comes to acute pancreatitis.

### 2.2. Etiologies

To understand the etiology of acute pancreatitis, obtaining a proper history upon initial admission is critical. The different etiologies include gallstones, alcohol abuse, autoimmune issues, smoking, hypertriglyceridemia, pancreas divisum, obesity, drugs, and pancreatitis post-endoscopic retrograde cholangiopancreatography (ERCP), and pancreatitis may even be multifactorial in certain scenarios.

#### 2.2.1. Gallstones

Gallstones are the leading cause of acute pancreatitis worldwide [8], and they account for ~50% of all cases in the West [9]. A few epidemiological studies have indicated an increasing prevalence of cholelithiasis with age [10]. Furthermore, there is a higher prevalence of gallstones in women, accounting for 66% of the cases of biliary pancreatitis [9]. Hence, patients hospitalized for acute pancreatitis almost always undergo a diagnostic right upper-quadrant ultrasound as part of the initial work-up to rule out gallstone pancreatitis and identify biliary duct dilatation that might even indicate cholangitis in certain cases requiring ERCP [11].

#### 2.2.2. Alcohol

Heavy alcohol use is a known cause of acute pancreatitis. It is the second most common cause of acute pancreatitis in North America and Europe, accounting for almost 33% of the cases [12,13]. Binge drinking means consuming five or more alcoholic drinks per session, corresponding to 70 g for men and 56 g for women. The impact of binge drinking is evidenced by the spike in cases of acute pancreatitis during the holiday season [14,15]. An important correlation observed for alcohol abusers is the pattern of drinking for more than several years prior to a pancreatitis episode [16]. Smoking is a big risk factor for acute pancreatitis, in addition to recurrent acute pancreatitis and chronic pancreatitis [17]. Low alcohol consumption (< two drinks/day for men and <one drink/day for women) may protect non-smokers from the first episode of pancreatitis, but not subsequent episodes of acute pancreatitis, by stimulating pancreatic ductal secretion [18,19].

#### 2.2.3. Hypertriglyceridemia

In a recent global systematic review, hypertriglyceridemia was determined to account for 9% of episodes of acute pancreatitis, making it the third most common cause [20]. A study at a high-volume tertiary care center in China reported that 33% of acute pancreatic episodes occurred secondary to hypertriglyceridemia [21]. The Endocrine Society classifies hypertriglyceridemia into mild (150–500 mg/dL), moderate (500–1000 mg/dL), or severe (>1000 mg/dL) forms [22]. There is an approximately 4% increase in the incidence of acute pancreatitis for every 100 mg/dL rise in serum triglyceride levels above 1000 mg/dL [23]. It is essential to order a lipid panel upon a patient’s admission to identify whether hypertriglyceridemia is the sole cause or a multifactorial cause of acute pancreatitis, as the prognosis is worse when it is the primary cause [24].

#### 2.2.4. Drugs

A thorough history and medicine reconciliation, including a history of consumption of any herbal supplements, are key to assessing patients presenting with acute pancreatitis. Although most drugs are reported to cause acute pancreatitis within one week of initiation, many drugs take weeks or months to trigger an acute attack of pancreatitis. Drugs account for ~5% of acute pancreatitis cases [25]. Drugs have been classified into four classes, with Class I drugs being defined as those featured in at least one case report describing the recurrence of acute pancreatitis following a drug challenge [26]. Some Class I drugs include antibiotics (tetracyclines and cotrimoxazole), steroids (prednisone, dexamethasone, and estradiol), antiepileptics (carbamazepine and valproic acid), anti-hypertensives (lisinopril, losartan, and furosemide), and opiates (codeine) [27]. There is some evidence suggesting the existence of intrinsic mechanisms of dose-dependent toxicity in some drugs and metabolites [28].

#### 2.2.5. Post-ERCP Pancreatitis

Based on the current literature, ERCP is associated with 1–2% of pancreatitis episodes. A systematic review of >100 randomized clinical trials showed that ~9% of episodes of acute pancreatitis constituted post-ERCP pancreatitis, and this figure ranged up to 14% for high-risk patients [29]. Multiple attempts at bile duct cannulation, unintended pancreatic duct cannulation, and prolonged procedures also increase the risk of post-ERCP pancreatitis. The patients at the greatest risk are young women with small bile ducts and sphincter of Oddi dysfunction [30,31].

#### 2.2.6. Other Causes of Acute Pancreatitis

Some rare causes of pancreatitis have been observed and need to be addressed to prevent recurrence and complications of acute pancreatitis. These include trauma, hypercalcemia, viral infections (coxsackie B virus, cytomegalovirus, mumps, and severe acute respiratory syndrome coronavirus 2 (SARS-CoV-2)), cardiac bypass surgery, and scorpion bites [32]. These causes can also further exacerbate episodes of acute pancreatitis. Treatment strategies usually involve supportive care but also depend on the underlying etiology. For instance, acute pancreatitis with a viral etiology will involve management of the viral infection concurrently with supportive care. In cases of cardiac-bypass-surgery-associated pancreatitis, the pathophysiology is thought to be ischemic, and these cases almost always improve once cardiac function improves [33]. In cases of scorpion bites, the local poison control authorities should be made aware, and an anti-venom can be used, although no large studies on this topic exist.

## 3. Disease Course

### 3.1. Pathophysiology

For the development of effective therapies to minimize pancreatic and systemic injuries, it is crucial to understand the critical mechanisms of acute pancreatitis [34]. The trigger event will induce an injury to pancreatic acinar and ductal cells by disrupting normal intracellular calcium signaling, hence affecting stimulus–secretion coupling [35]. In the case of high toxin exposure, there is a greater trigger, further damaging the pancreatic cells and diminishing ATP production. This results in defective autophagy, the localization of endolysosomes, inflammasome activation, cytokine release, and cellular necrosis [34,36].

**Figure 2:**
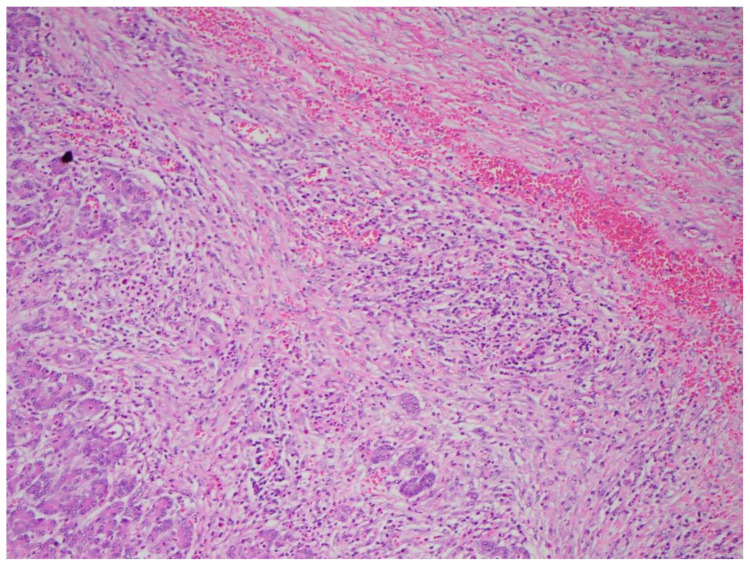
Acute pancreatitis with hemorrhaging (H&E stain, 10×).

**Figure 3:**
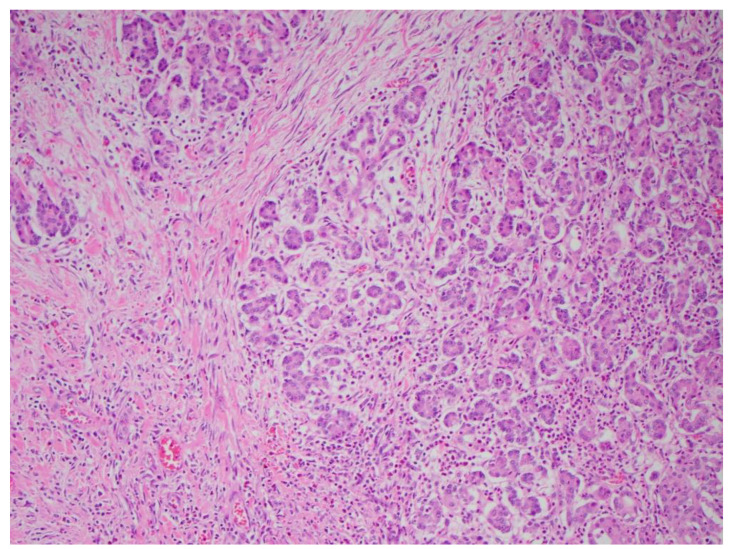
Acute pancreatitis as seen at 10× magnification (H&E stain).

Multiple cytokines mediate a powerful pro-inflammatory immune response, such as tumor necrosis factor-alpha (TNF-a) and interleukins 1a, 1b, 6, and 18, exacerbating the initial pancreatic injury [37]. Pathologically, this appears as inflammation and can also be associated with a hemorrhage at the microscopic level (Figure 2 and Figure 3). The cytokine-mediated inflammatory cascade then extends the inflammatory cascade via lymphatic and systemic circulation into the liver, lungs, heart, kidneys, and gastrointestinal (GI) tract, leading to multi-organ injury [38]. This can cause systemic inflammatory response syndrome (SIRS), an early clinical feature that persists in cases of severe acute pancreatitis. Inflammation and damage to the GI tract can lead to bacterial translocation [39], and the species of bacteria involved are a predictive factor of disease severity, with Enterococcidae most frequently being associated with severe disease [40]. Obesity is also a predictive factor for moderate severity, and it leads to further deleterious effects through adipocyte lipolysis in the pancreas and adipose tissue [41,42] (Figure 2 and Figure 3).

### 3.2. Severity of Pancreatitis

**Figure 4:**
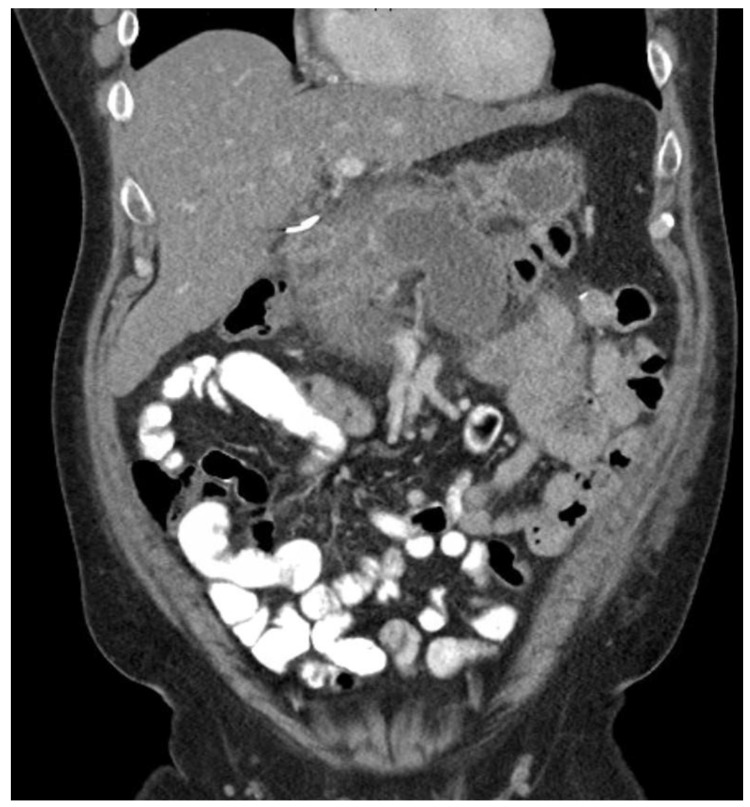
Pancreatitis with CT showing walled-off necrosis.

**Figure 5:**
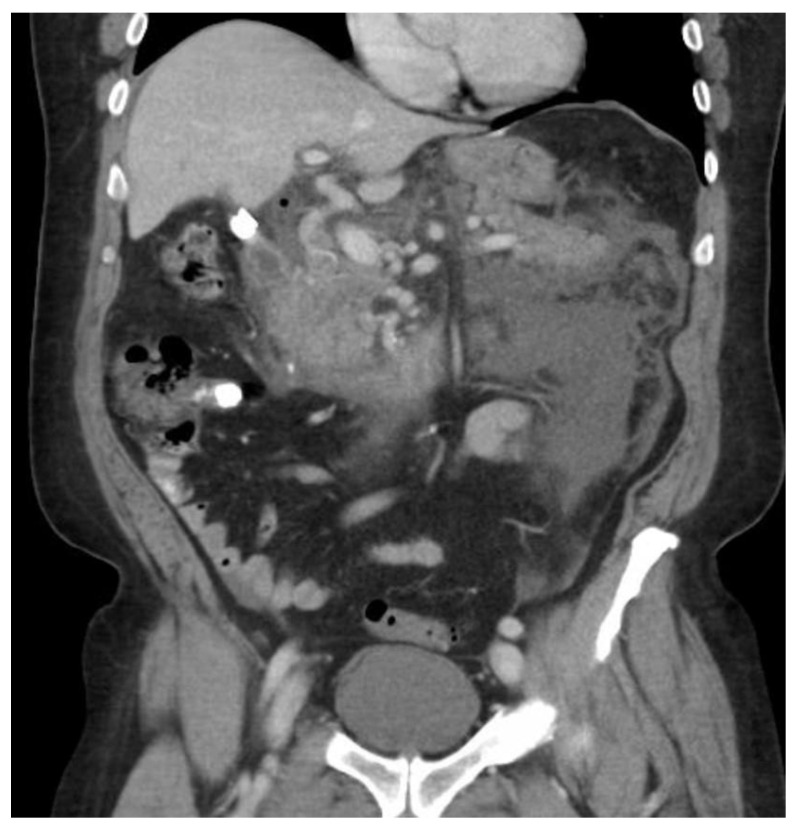
Acute interstitial pancreatitis with developing peripancreatic fluid collection.

**Figure 6:**
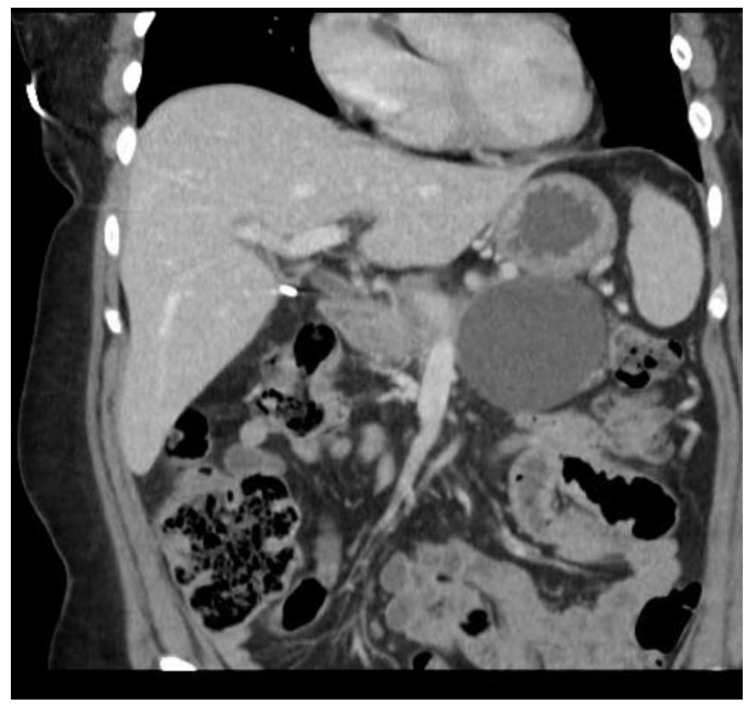
Pseudocyst of the pancreas.

The prediction of severity is made upon the admission of the patient, but the actual degree of severity is determined once sufficient time has elapsed in order to make a better assessment of acute pancreatitis. The most widely accepted classification of severity is the Revised Atlanta Classification (RAC) [4], which classifies pancreatitis as follows: (1) mild acute pancreatitis with no local inflammation or organ failure, (2) moderately severe acute pancreatitis with transient organ failure (<48 h) or local complications, and (3) severe acute pancreatitis with persistent organ failure (>48 h). In total, 65–70% of patients with acute pancreatitis have an uncomplicated course in which the symptoms resolve within a few days [4,43]. A total of 20–25% of patients develop moderate acute pancreatitis with local pancreatic injury fluid collection or necrosis, leading to prolonged hospitalization. This progression is displayed in Figure 4, Figure 5 and Figure 6. About 10% of patients develop severe acute pancreatitis accompanied by severe pain, a nutritional deficit, and a hospital stay > 4 weeks. These patients require highly intensive critical care with interventions (as indicated) [24].

An alternative classification scheme is Determinant-based Classification (DBC) [43], with four categories: mild—no necrosis or organ failure; moderate—sterile necrosis or transient organ failure (<48 h); severe—infected necrosis or persistent organ failure (>48 h); and critical—infected necrosis and persistent organ failure [44]. Interestingly, recent evidence has established that infected pancreatic necrosis has a lesser impact on mortality [45]. While these two classifications provide greater insight into the course of acute pancreatitis, the optimal categorization remains elusive.

One of the key limitations of classification systems for pancreatitis severity is the need to sometimes wait greater than 24 h prior to classification. It is well known that there is irreversible leakage of plasma proteins through endothelial cells during an episode of AP. Identifying patients that may develop this capillary leak may be crucial to preventing multi-organ failure in cases of severe pancreatitis [46].

Furthermore, studies have also shown that albumin levels exhibit a good correlation with predicting multi-organ failure in AP [47,48].

In the age of personalized medicine, the genetic blueprint of an individual may also help in determining the severity of pancreatitis. The previously published literature suggests there is some correlation between specific gene associations and the severity of pancreatitis [49]. However, the data on this aspect are limited, and covering this in detail is beyond the scope of this review; however, it is completely conceivable that this may represent a critical topic in the future.

### 3.3. Acute Pancreatitis in the Elderly

The incidence of gallstones increases with age [50], with biliary pancreatitis being the most common etiology for elderly patients [51]. Frailty and co-morbidities increase the likelihood for less favorable outcomes of acute pancreatitis [52]. There is a high rate of idiopathic pancreatitis (30–40%) in the elderly population [53] despite the availability of modern imaging and advanced endoscopy. Polypharmacy can lead to drug-induced pancreatitis, which often goes unnoticed. There is also a significant risk of malignancy in this group, wherein pancreatitis occurs secondary to an obstruction of the pancreatic head by tumors [54]. Autoimmune pancreatitis is also common in elderly patients, so serum IgG4 should be considered [55].

### 3.4. Acute Pancreatitis in Children

The incidence of acute pancreatitis is lower in children, but the diagnosis criteria remain the same. The most common symptoms are abdominal pain, distension, nausea, and vomiting. Transabdominal ultrasound is the preferred imaging modality [56] in order to prevent contrast exposure, and large randomized controlled large-center studies are needed to increase the ability to better delineate pancreatitis and its management and complications.

### 3.5. Acute Pancreatitis in Pregnancy

Acute pancreatitis occurs in around 1 in 5000 pregnancies, a figure higher than that for the general population [57], probably due to hormonal changes affecting bile flow, leading to greater gallstone formation [58]. A cohort study of 31,494 women found a relative risk of 1.57 for acute pancreatitis in those receiving hormone replacement therapy [59]. Moreover, hypertriglyceridemia-associated acute pancreatitis has been linked to estrogen therapy for fertility treatment [60,61]. Fortunately, the outcomes for patients and fetuses have substantially improved over the last few decades, a fact largely attributed to improvements in technology, advanced imaging modalities (such as endoscopic ultrasound), and early diagnosis [59].

## 4. Inpatient Management of Acute Pancreatitis

Patients admitted for acute pancreatitis are monitored regularly for oxygen requirements and vital signs. The initial investigation includes serum amylase and lipase analysis, a lipid panel including triglycerides, a full blood count, a complete metabolic panel, an analysis of electrolytes, a hemoglobin A1 c test, and transabdominal ultrasound. The initial therapy includes oxygen, intravenous fluid provision, pain control, and a nutrition regime.

### 4.1. Oxygen

An oxygen saturation (SpO2) degree of 94–99% is an appropriate target range for oxygen, and inspired oxygen delivery system, flow rate, and saturation data should be regularly recorded. Lower rates of 88–92% are appropriate for patients with chronic obstructive pulmonary disease (COPD) or morbid obesity. If initial saturation is <85%, 1 L/min of oxygen provided via a reservoir mask should be administered and reduced if the patient stabilizes [62]. Arterial blood gas measurements should be utilized if there is a desaturation episode, and oxygen requirements should be adjusted as indicated.

### 4.2. Intravenous Fluid Resuscitation

Appropriate administration of intravenous fluids is key in the management of acute pancreatitis within 24 h of its onset. This results in lower rates of SIRS and organ failure, and the recommend rate is 5–10 mL/kg/h [24]. This corrects third-space volume loss and tissue hypoperfusion counteracting pancreatic and systemic microcirculatory impairment of the inflammatory cascade levels [63]. With fluid resuscitation, the aim is to decrease a patient’s heart rate below 120 beats/min and increase their urine output > 0.5 mL/kg/h. We need to be cautious and prevent excessive fluid intake leading to volume overload in critical care settings as this is associated with an increased risk of death due to negative effects on all major organ systems [64]. One trial involving 40 patients suggested lactated ringer’s solution is preferable to saline in the initial phase of resuscitation [65], and these findings were confirmed in a subsequent smaller trial [66]. Further research is necessary to improve strategies for each phase of fluid therapy: resuscitation, optimization, stabilization, and evacuation (ROSE) [64].

### 4.3. Pain Control

Randomized trials have demonstrated that opiates are safe and effective for pain control initially [67], and then NSAIDs can be used as an alternative but should be avoided in cases involving renal damage [68]. Early oral nutrition provision without worsening abdominal pain may help reduce pain intensity and duration [69]. The intensity and duration of pain are broadly proportional to disease severity and total opiate administration [70]. The management of pain and psychological stress associated with acute pancreatitis are interdependent and should be given equal importance. Psychological support should be offered to patients to build a healthier relationship and thus provide them with confidence and reduce their anxiety levels.

### 4.4. Nutrition

Acute pancreatitis induces a hypermetabolic state (involving a reduction in body mass, insulin resistance, protein catabolism, and lipolysis), which is exacerbated by inadequate nutrition and infections [71]. Hence, early oral feeding is encouraged if *tolerated*; if not, liquid supplements or enteral tube feeding within 48 h of admission is recommended [72]. It is important to realize that oral or enteral feeding prevents bacterial translocation across the GI permeability barrier and reduces pro-inflammatory responses [73]. In cases of inadequate nutrition, the combined use of enteral and parenteral nutrition should be considered, but current trials and meta-analyses do not show that this combined approach is superior to oral or enteral approaches [74].

## 5. Complications of Acute Pancreatitis

### 5.1. Necrosis and Infection Management

In cases of severe abdominal pain for more than 3 days, it is important to further evaluate a patient’s condition using inflammatory markers: C-reactive protein {CRP) and erythrocyte sedimentation rate (ESR). For imaging, a CT scan is initially appropriate. Timely identification of local complications is crucial, and for pancreatic necrosis or fluid collection, a drain should first be placed endoscopically or percutaneously [75]. If signs of infection or gas are present in the CT scan, it is important to expedite drainage placement or even perform a necrosectomy with proper antibiotic usage if bacterial translocation has taken place.

Over the past several years, there has been an emphasis on adopting a step-up approach to the management of infected necrotic collections in the context of AP. The concept is fairly straightforward and primarily advocates for minimally invasive interventions prior to the adoption of more aggressive surgical options, leading to improved overall outcomes.

A landmark controlled trial conducted by van Santvoort and co-workers provided excellent evidence for the step-up approach. They studied the outcomes of patients who were subjected to a step-up approach to open necrosectomy and found this approach was associated with a significant decrease in morbidity and mortality [76]. This pivotal trial changed the entire paradigm of the management of necrotic collections.

Another landmark study from 2019 evaluated outcomes of early versus late catheter drainage in cases of infected necrotizing pancreatitis. This study added another layer of understanding to our knowledge of the management of these complex collections. Specifically, the researchers found that the choice of timing should be tailored to the individual patient’s clinical condition [77].

Several recent review articles on this topic have been published, covering the indications of endoscopic necrosectomy, the timing of interventions, when to use a percutaneous and/or endoscopic approach, and the benefits of a multi-disciplinary approach [78,79].

To summarize, the management of necrotic pancreatic collections is ideally tailored to a specific patient and conservative. Furthermore, a step-up approach is preferred to reduce morbidity and mortality and shorten hospital stays.

### 5.2. Acute Compartment Syndrome

One of the most feared complications of AP is acute compartment syndrome. Acute compartment syndrome (ACS) can arise from severe AP wherein peripancreatic and intra-abdominal edema results in increased intra-abdominal hypertension and elevated intra-abdominal pressure leading to a decrease in the perfusion of vital organs, leading to multi-organ failure. Inflammation and fluid accumulation lead to the over-distention of the abdominal cavity and subsequently ACS [80]. Early recognition and intervention are key in the management of compartment syndrome, particularly ACS [81]. Surgical interventions, especially minimally invasive techniques, have also evolved over the years. Specific surgical management is beyond the scope of this review, although the reader can refer to other articles on this topic [82,83,84].

### 5.3. Diabetes Mellitus Management

Due to repeated episodes of acute pancreatitis, the scarring of pancreas parenchyma takes place, affecting insulin production capacity, leading to the development of insulin resistance. Tu et al. reported that the chance of developing impaired glucose tolerance was ~ 60% in the 5 years following the first attack of acute pancreatitis [85]. Patients who undergo a necrosectomy have an even greater risk of developing diabetes. For patients who experience hypoglycemia episodes on diabetes medicine, a continuous subcutaneous insulin pump is an alternative [86].

### 5.4. Pancreatic Enzyme Replacement Therapy

Pancreatic exocrine insufficiency can be seen after acute pancreatitis episodes in >50% patients, but the frequency falls in subsequent months. If the patient develops pancreatic necrosis of >50%, then pancreatic enzyme supplementation would be necessary, for which either commercially available Creon^®^ or Zenpep^®^ can be used. Both formulations have pancreatic lipase, which is a key enzyme that aids in digestion. It is also prescribed for moderately severe and severe acute pancreatitis with oral feeding until fecal elastase values are consistently normal (>200 µg/g) [87]. The normal human adult pancreas secretes between 1 and 2 million units of lipase daily along with many proteases, carbohydrate hydrolases, lipid hydrolases, and nucleases [88]. The minimum recommended standard dose of pancrealipase is 50,000 units with a meal, and half of that with a snack, and up-titration is recommended based on the steatorrhea level.

## 6. Prevention of Recurrence of Pancreatitis

**Figure 7:**
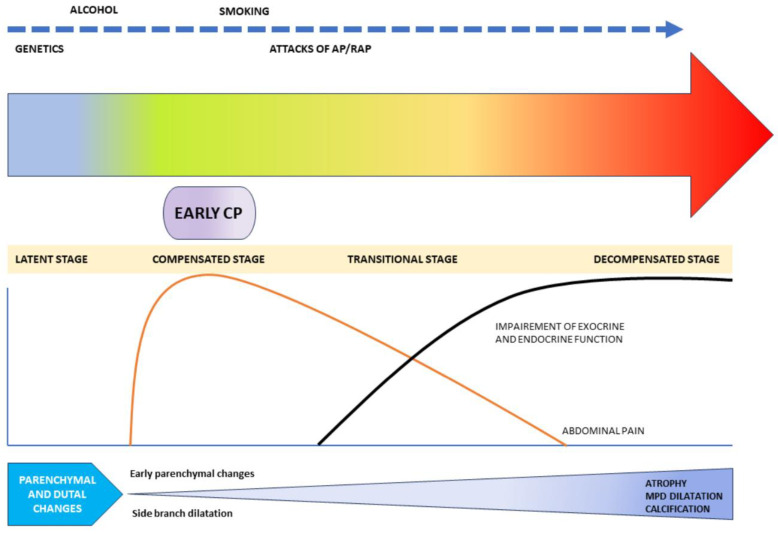
Schematic representation of the progression of pancreatitis from acute to chronic.

After an initial attack of acute pancreatitis, about 20% of patients have a recurrence, and half of them develop chronic pancreatitis, especially males who consume alcohol and smoke [89]. To prevent alcohol- or smoking-related pancreatitis, abstinence is needed. An Icelandic study observed recurrent acute pancreatitis attacks were suffered by one-third of persistent drinkers, while none were suffered by abstainers, over 5 years of follow-ups [90]. In addition, the most effective method of preventing recurrent biliary pancreatitis is cholecystectomy. The aim in the prevention of acute pancreatitis associated with recurrent episodes of hypertriglyceridemia is to reduce serum triglyceride levels at least below 1000 mg/dL (ideally below 500 mg/dL). The first line of therapy is lifestyle changes (exercise, diet control, and omega-3 fatty acids), followed by lipid-lowering medications (fibrates with or without statins) [22]. In treatment-resistant hypertriglyceridemia, plasmapheresis may help reduce recurrence [91]. For drug-induced acute pancreatitis, the offending agent needs to be stopped immediately, and later resumption of a drug would depend on if other alternatives are not successful. For autoimmune pancreatitis, the recommended procedure is oral prednisolone administration (2 mg/kg, with a maximum of 60 mg daily), tapered slowly by 5–10 mg weekly, staying on a maintenance dose (5 mg/day) for 6 months with close follow-ups [92]. Therapy can be escalated to rituximab or azathioprine in case of steroid resistance or relapsing disease [93]. Lastly, patients with pancreas-sufficient cystic fibrosis are at risk of acute pancreatitis, which can be reduced by cystic fibrosis transmembrane conductance regulator (CFTR) modulator therapy, such as with Ivacaftor or Tezacaftor [94]. Hence, for each cause of acute pancreatitis, there are strategies available to prevent recurrent episodes with a proper step-by-step approach. An excellent schematic showing the progression of pancreatitis from acute to chronic over time that was previously created by the author of this article is shown below (Figure 7) [95].

## 7. Conclusions

The mortality rate of acute pancreatitis has decreased in the past few decades because of better management skills, but the recent rise in acute pancreatitis episodes is concerning. Acute pancreatitis is a common disease affecting all age groups, with variations in etiologies. The initial management remains supportive, involving oxygen and fluid supplementation, pain control, and the optimization of the patient’s nutrition status. Proper care should be taken to determine the cause of acute pancreatitis to prevent recurrent episodes. Sustained efforts are required through clinical trials to establish a broad variety of drugs that can be used for acute pancreatitis episodes.
